# Severe Ptyalism from a Dental Operation without Amalgam

**Published:** 1872-04

**Authors:** A. Berry


### Communications.

### SEVERE PTYALISM FROM A DENTAL OPERATION WITHOUT AMALGAM.

BY A. BERRY, D. D. S.

Some time ago in my early practice a lady requested me to insert for her two central incisors, the crowns of which were lost several years previously. The gums and mouth being very much diseased, I declined to comply with her wishes, assigning as a reason the danger of inflammation. But several months subsequently, the condition of the mouth being improved, I erred like my professional confrere in Kansas recently (although from different reasons my judgment being influenced by desire to gratify my patient, and his, it seems from the report, from alcoholic potations) and prepared the roots for artificial crowns, which were fitted with pivots in them but not inserted, as I thought best to defer that part of the operation to see what would turn up.

In a few hours my patient was enduring the ills of enormous swelling of her entire face, severe pain and excessive salivation. Calling her physician promptly he found her giving expressions not complimentary to her dentist; but being a gentleman, he assured the lady that her sufferings were not to be charged to the fault of the dentist, but that the salivation and swelling, which was so extensive that deglutition was performed with difficulty, were simply a recurrence induced by irritating the diseased teeth of a severe and dangerous ptyalism some ten or twelve years before, the marks of which she still bore on her neck. A week elapsed before I saw her, when she assured me that in the meantime she kept her bed with saliva flowing constantly from her mouth during four days. When the periostitis subsided, as I was not at hand, she put the teeth in place and wore them about seven years.

Had there been an amalgam filling in the patient's teeth and the case terminated fatally, like that reported lately in Kansas, it might have afforded ground for persons wise above what is known to have originated an alarming tale of "another death from bad dentistry."

